# Beta-cell regeneration from vimentin^+^/MafB^+^ cells after STZ-induced extreme beta-cell ablation

**DOI:** 10.1038/srep11703

**Published:** 2015-07-01

**Authors:** Yu Cheng, Hongjun Kang, Jing Shen, Haojie Hao, Jiejie Liu, Yelei Guo, Yiming Mu, Weidong Han

**Affiliations:** 1 Department of Endocrinology, Chinese PLA General Hospital, 28 Fuxing Road, Beijing 100853, China; 2 Department of Molecular Biology, Institute of Basic Medicine, School of Life Science, Chinese PLA General Hospital, 28 Fuxing Road, Beijing 100853, China; 3 Department of Endocrinology, Chinese PLA 309 Hospital, 17 Heishanhu Road, Beijing 100091, China; 4 Department of Critical Care Medicine, Chinese PLA General Hospital, 28 Fuxing Road, Beijing 100853, China

## Abstract

Loss of functional beta-cells is fundamental in both type 1 and type 2 diabetes. *In situ* beta-cell regeneration therefore has garnered great interest as an approach to diabetes therapy. Here, after elimination of pre-existing beta cells by a single high-dose of streptozotocin (STZ), we demonstrated that a considerable amount of beta-like-cells was generated within 48 hrs. But the newly formed insulin producing cells failed to respond to glucose challenge at this time and diminished afterwards. Insulin treatment to normalize the glucose level protected the neogenic beta-like cells and the islet function was also gradually matured. Strikingly, intermediate cells lacking epithelial marker E-cadherin but expressing mesenchymal cell-specific marker vimentin appeared within 16 hrs following STZ exposure, which served as the major source of insulin-producing cells observed at 24 hrs. Moreover, these intermediate cells strongly expressed alpha-cell-specific marker MafB. In summary, the data presented here identified a novel intermediate cell type as beta-cell progenitors, showing mesenchymal cell feature as well as alpha-cell marker MafB. Our results might have important implications for efforts to stimulate beta-cell regeneration.

Diabetes has become a major public healthcare problem in the world. Loss of functional β-cells is fundamental in both type 1 and type 2 diabetes^1^,^2^. A therapeutic ideal—relative to pancreas and islet transplantation—would be to stimulate a resident source, thus avoiding the caveats of limited graft survival, donor shortage and host immune rejection^3^,^4^,^5^. The ability of the pancreas to generate new beta-cells has been described in a number of models where pancreatic injury have been developed, including chemical and genetic beta-cell ablation, partial pancreatectomy, and pancreatic duct ligation (PDL)^6^,^7^,^8^,^9^. The regeneration processes could be induced by replication of pre-existing beta-cells, neogenesis from endogenous progenitors or transdifferentiation from differentiated non-beta cells, revealing a surprising degree of cell plasticity in the mature pancreas. Using a strategy of re-expressing key regulators of beta-cell developmental *in vivo* (Ngn3, Pdx1, MafA), differentiated pancreatic exocrine cells in adult mice were reprogrammed into cells that closely resemble beta-cells^10^, and the lineage-reprogrammed cells survived and functioned *in vivo* over a long term^11^.

According to previous reports, extreme beta-loss in adults appears to trigger reprograming of alpha-cells into beta-cells. In a transgenic model of diphtheria-toxin-induced acute selective near-total beta-cell ablation without inflammation or autoimmunity, large fractions of regenerated beta-cells are derived from alpha-cells^8^. Interestingly, using the exact same model, extreme beta-loss before puberty induces the spontaneous en masse reprogramming of somatostatin-producing delta-cells to beta-cells^12^.

Streptozotocin (STZ) preferentially accumulates in pancreatic beta-cells via the Glut2 glucose transporter, fragments DNA and therefore specifically destroys beta-cells in pancreas^13^,^14^. A single high dose of STZ-induced diabetic model is routinely used in diabetic research, which also resulted in near-total ablation of beta-cells^15^. Consistently, regeneration and diabetes recovery in juvenile mice after inducing beta-cell destruction with STZ are also delta-cell-dependent^12^. However, beta-cell regeneration has never been reported in single high dose STZ-treated adult rodents.

Here, after careful examination by sacrificing rats at different times since very soon following a single high dose of STZ, we observed rapid beta-cell regeneration within 48 hrs after extreme loss of beta-cells, with neogenic beta-cell number accounting for about 14% of the normal control. The regenerated beta-cells survived and acquired functionality over time with insulin treatment. A surprisingly large proportion of newborn insulin^+^ cells at 24 hrs after STZ-treatment co-expressed with vimentin while did not show typical mesenchymal cell shape but were round-shaped. More importantly, we detected very strong expression of MafB, an alpha-cell specific marker in adult rodents, in the vimentin^+^/insulin^+^ cells.

## Results

### Ablation of beta-cells after a single high dose of STZ injection

First, we sought to determine whether STZ eliminated almost all beta-cells in islets post STZ injection. Examination of serial sections of pancreas stained with insulin revealed that almost all the beta-cells lost their clear cytoplasmic compartments at 8 hrs after STZ injection and the islets were occupied with cell debris and scattered nuclei (Supplementary Fig. 1A). At this time, the pancreas was massively infiltrated by macrophages engulfing the necrotic cells (Supplementary Fig. 1B). At 16 hrs, the stained cell debris was virtually cleared. Consistently, hematoxylin and eosin staining of islets showed that the cytoplasm of almost all the beta-cells were faintly stained by eosin and the nuclei were pyknotic 8 hrs post STZ injection, and the beta-cell area was hollowed at 16 hrs except for some round-shaped cells (Supplementary Fig. 1C). As the presence of stained cell debris could hinder the detection of residual cells in the islet, we counted the beta-cell number 16 hrs post STZ treatment; only 12.5 ± 0.8 stained cells were observed per islet, accounting for less than 1% of the normal beta-cell number (Supplementary Fig. 1D). In addition, the insulin transcription level had dropped to 0.2% of the control value (Supplementary Fig. 1E,F). Taken together, all the data showed that a single high dose of STZ induced acute and extreme beta-cell loss in adult rats.

### Rapid beta-cell regeneration following STZ treatment

**Figure 1:**
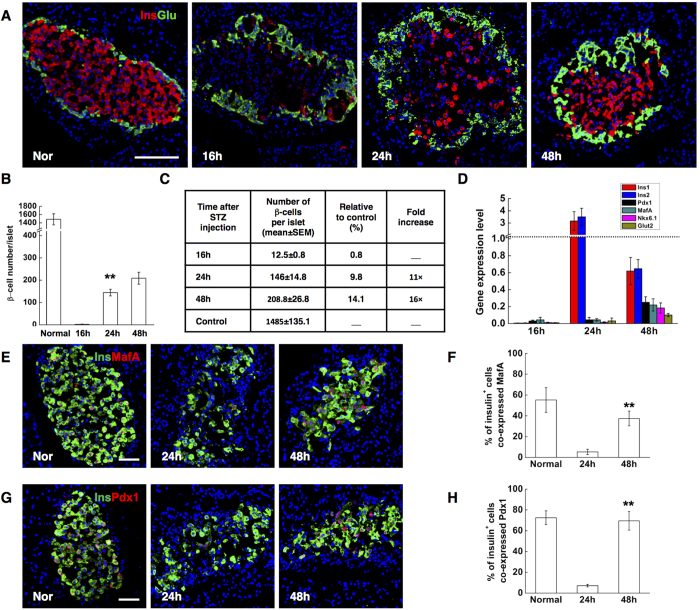
STZ treatment induced rapid beta-cell neogenesis in adult rat pancreas (**A**) Representative islets stained with antibodies against insulin (red) and glucagon (green) of normal control SD rats (Nor), and STZ-treated rats at indicated time points (16 h, 24 h, 48 h,). Nuclei were labeled with DAPI. Scale bars, 100 μm. (**B**,**C**) Quantification of beta-cell number. (**D**) Expression of beta-cell enriched genes in isolated islets of normal control and STZ-treated rats at indicated time points (16 h, 24 h, and 48 h). (**E**) Photomicrographs double stained with anti-insulin (green) and anti-MafA (red) antibodies from normal control rats (Nor), and STZ-treated rats (24 h, 48 h). Nuclei were labeled with DAPI. Scale bars, 50 μm. (**F**) Quantification of insulin positive cells co-expressing MafA. (**G**) Photomicrographs double stained with anti-insulin (green) and anti-Pdx1 (red) antibodies. Nuclei were labeled with DAPI. Scale bars, 50 μm. (**H**) Quantification of insulin positive cells co-expressing Pdx1. Data are shown as means ± SEM, n = 5–6 rats per group; *P < 0.05 and **P < 0.01.

To explore the possibility of beta-cell regeneration and its kinetics, rats were sacrificed at different time points after beta-cell ablation. Surprisingly, between 16 hrs and 24 hrs following STZ injection, the number of insulin-stained cells in each islet increased by a factor of 11 (from 12.5 ± 0.8 to146 ± 14.8). At 48 hrs, the insulin^+^ cell number further increased to 209 per islet, corresponding on average to 14.1% of the normal beta-cell number (Fig. 1A–C). In fact, 60% of islets contained no more than ten insulin-stained cells per islet 16 hrs after ablation, whereas at 48 hrs, almost all medium and large islets showed signs of beta-cell regeneration. 96% of medium and large islets contained more than 100 insulin^+^ cells. No beta-cells were found in extra-insular locations. Next, we detected the expression of key beta-cell transcription factors MafA and Pdx1 by immunofluorescence. Almost all of the insulin^+^ cells at 24 hrs lacked detectable levels of MafA and Pdx1 (Fig. 1E,G). At 48 hrs, the ratio of insulin^+^ cells expressing Pdx1 significantly increased up to 69.5% (Fig. 1F), showing no difference with the normal control, and the ratio for MafA expression also elevated to 37.4% (Fig. 1H), although still slightly lower than normal level. These observations indicate that rapid beta-cell regeneration occurred after STZ-induced near-total beta-cell destruction.

To confirm our immunohistochemistry results, we conducted quantitative RT-PCR analysis for key beta-cell genes. Several beta-cell markers were severely down-regulated after beta-cell loss, as expected. Interestingly, at 24 hrs, transcription of two insulin genes increased by a factor of 1500, even up to 3 fold of the normal control, suggesting drastic activation of the two genes during the initial period. Other beta-cell-enriched genes, which were not activated at 24 hrs, were markedly unregulated at 48 hrs (Fig. 1D).

### Neogenic beta-cells survived and acquired functionality after insulin treatment

In long-term experiments, however, these neogenic cells rapidly diminished during the next few days. Prolonged exposure to high concentration of glucose induced beta-cell deterioration and apoptosis^16^,^17^,^18^. At 48 hrs, the newborn insulin-producing cells seemed insufficient to control the blood glucose level as the animals maintained severe hyperglycemia (Supplementary Fig. 2), which probably resulted in the disappearance of regenerated beta-cells. We tested this suggestion by treating rats with a long-acting human insulin analogue for 7 days and comparing the outcome relative to non-treated rats.

**Figure 2:**
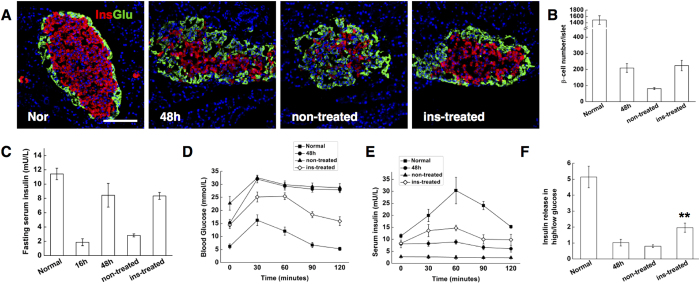
Neogenic beta-cells survived and acquired functionality after insulin treatment. (**A**) Representative islets stained with antibodies against insulin (red) and glucagon (green) of normal control SD rats (Nor), STZ-treated rats after 48 hrs (48 h), ins-treated rats after 7 days’ insulin treatment with a one-day washout (insulin-treated), and concurrent non-treated control rats (non-treated). Nuclei were labeled with DAPI. Scale bars, 100 μm. (**B**) Quantification of beta-cell number. (**C**) Fasting serum insulin levels in normal control SD rats (Nor), STZ-treated rats after 16 and 48 hrs (16 h, 48 h), insulin-treated rats by day 7 (insulin-treated) and concurrent non-treated control rats (non-treated). (**D** and **E**) Plasma glucose and insulin during an IP-GTT. (**F**) GSIS *in vitro*, measured as the ratio of insulin release in high glucose to insulin release in low glucose. Data are shown as means ± SEM, n = 6 rats per group; **P < 0.01.

Using insulin to normalize blood glucose level appeared to stabilize the beta-cell number, which accounted for approximately 15% of the normal control at the end of observation, while the number of insulin stained cells of non-treated rats dropped to 5.4% (Fig. 2A,B). ELISAs on blood demonstrated that fasting serum rat insulin level increased by a factor of 4 (from 1.86 ± 0.48 to 8.43 ± 1.65 mU/L) from 16 hrs to 48 hrs after STZ treatment, but significantly decreased to 2.8 mU/L 7 days later. The insulin therapy, on the other hand, helped to maintain the fasting serum insulin level (Fig. 2C). The data suggested that hyperglycemia was the main cause of the decreased beta-cell number.

**Figure 3:**
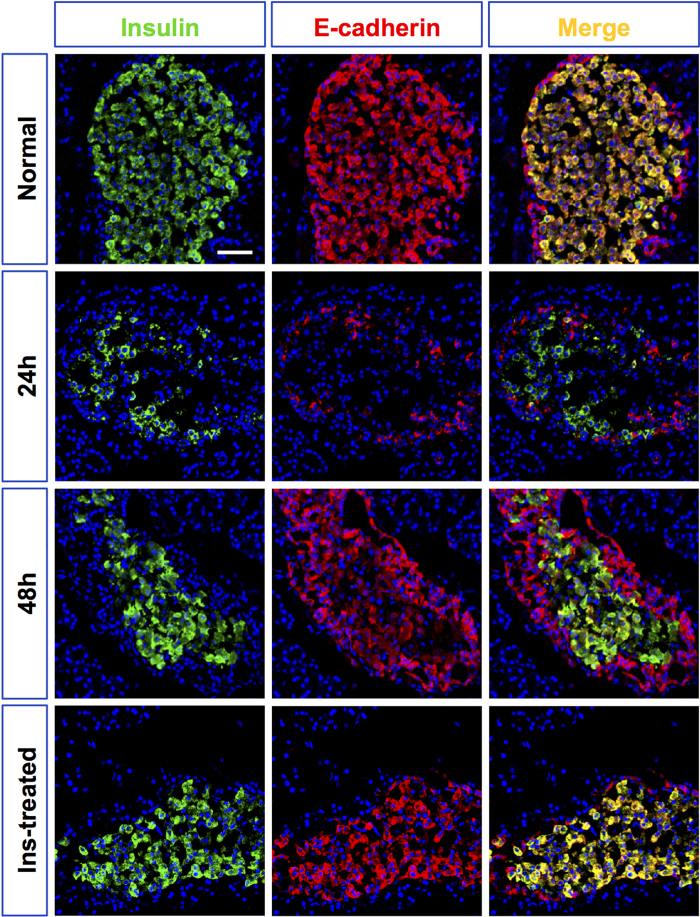
Temporal changes of E-cadherin expression in insulin^+^ cells following STZ treatment. The photomicrographs in the left column showed representative islets stained with anti-insulin (green), and the middle column with anti-E-cadherin (red) antibodies from normal control SD rats (Nor), STZ-treated rats at different time points (24 h, 48 h), and insulin-treated rats by day 7 (insulin-treated). The right column showed the merged images of the left and middle column. Nuclei were labeled with DAPI. Scale bars, 50 μm.

Next, we examined how the functional properties of the new insulin^+^ cells changed. The glucose responsiveness, a key measure of beta-cell function, was interrogated by IP glucose tolerance tests (IPGTT) and insulin release tests (IRTs) *in vivo* as well as glucose-stimulated insulin secretion (GSIS) tests *in vitro*. The newborn beta-cells at 48 hrs were profoundly glucose intolerant, with no change in plasma insulin in response to the glucose load. Consistently, no glucose responsiveness was detected *in vitro* (high glucose/low glucose response ratio of 1.028 ± 0.21). Functional beta cells were observed from animals after insulin treatment. By 7 days, regenerated insulin^+^ cells demonstrated improved glucose disposal, with increased GTT-stimulated plasma insulin level (Fig. 2D,E), as well as glucose-stimulated insulin secretion (high glucose/low glucose response ratio of 1.96 ± 0.28) (Fig. 2F). By contrast, the function of beta-cells in non-treated animals did not show any improvement over time (high glucose/low glucose response ratios of 0.80 ± 0.09). Inter-islet communication is important for beta-cell function^19^,^20^. To investigate whether the functionality of beta-cells was enhanced by cell-cell contact, the expression of the cell surface adhesion protein E-cadherin, which regulates adhesive properties of beta-cells^21^,^22^, was studied by immunofluorescence on pancreas sections. In normal islets, as previously reported^23^, E-cadherin was clearly expressed at the surface of beta-cells. Nonetheless, the neogenic insulin^+^ cells observed 24 hrs after STZ treatment remained as scattered individual cells or small clusters and did not aggregate together; less than five insulin-stained cells per islet at this time show E-cadherin staining. At 48 hrs, almost all the beta-like-cells gained E-cadherin expression but the expression level was much lower than that of the normal control. Beta-cells from insulin-treated rats, however, manifested strong expression of E-cadherin that was comparable with that of the normal control (Fig. 3).

Together, these studies indicate that regenerated beta-like cells survived, and gradually acquired enhanced cell-ell contact and functionality after reversing the hyperglycemic state by insulin treatment.

### A group of round-shaped vimentin^+^ cells replicated and differentiated into beta-like cells

**Figure 4:**
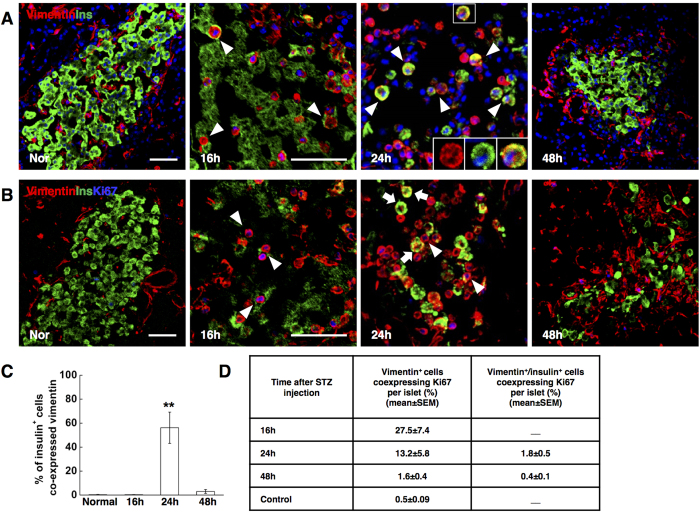
Neogenic beta-cells manifested vimentin expression. (**A**) Photomicrographs double stained with anti-insulin (green) and anti-vimentin (red) antibodies from normal control rats (Nor), and STZ-treated rats at different time points (16 h, 24 h, 48 h). Nuclei were labeled with DAPI. Scale bars, 50 μm. The isosceles triangle: insulin^+^ cells co-expressing vimentin. The boxed regions showed the high power photomicrograph at the bottom right of the image. (**B**) Photomicrographs triple stained with anti-vimentin (red), anti-insulin (green) and anti-Ki67 (blue) antibodies. The isosceles triangle: vimentin^+^ cells co-expressing Ki67. The arrows: vimentin^+^/insulin^+^ cells showing negative Ki67 expression. Scale bars, 50 μm. (**C**) Quantification of insulin positive cells co-expressing vimentin. (**D**) Quantification of vimentin^+^ and vimentin^+^/insulin^+^ cells co^-^expressing Ki67. Data are shown as means ± SEM, n = 5–6 per group; *P < 0.05 and **P < 0.01.

E-cadherin is also a crucial caretaker and molecular marker of the epithelial phenotype^24^,^25^. The lack of E-cadherin expression in insulin-staining cells at 24 hrs implied that they might exhibit mesenchymal cell properties. We then examined the expression of vimentin, a canonical mesenchymal marker, on insulin^+^ cells. A group of vimentin^+^ cells emerged 16 hrs after STZ treatment. These vimentin^+^ cells showed very specific morphological features; distinctive from mesenchymal cells, they manifested a typically round-shape. Intriguingly, at 24 hrs, the majority of insulin^+^ cells (about 60%) was co-expressed with vimentin and was round-shaped (Fig. 4A,C), revealing the group of vimentin^+^ cells as the major source of the new beta-like cells. At 48 hrs, almost all the beta-like cells lost vimentin expression and were morphologically identical to the normal beta-cells. During beta-cell reconstitution, between 16 hrs to 24 hrs following STZ injection, a high rate of the round-shaped vimentin^+^ cells (27.5 ± 7.4% to 13.2 ± 5.8%) proliferated. But in contrast, the round-shaped vimentin^+^ cells coexpressing insulin had a very low replication rate (1.8 ± 0.5%), suggesting that the reconstituted insulin cells were like beta-cells with very slow turnover in adults^26^ (Fig. 4B,D).

The results presented here suggested that massive beta-cell loss triggered a group of vimentin^+^ cells with unique morphology, which replicated, gradually gained insulin expression and subsequently, a beta-cell-like identity.

### Spared beta cells did not contribute to the rapid beta-cell regeneration

**Figure 5:**
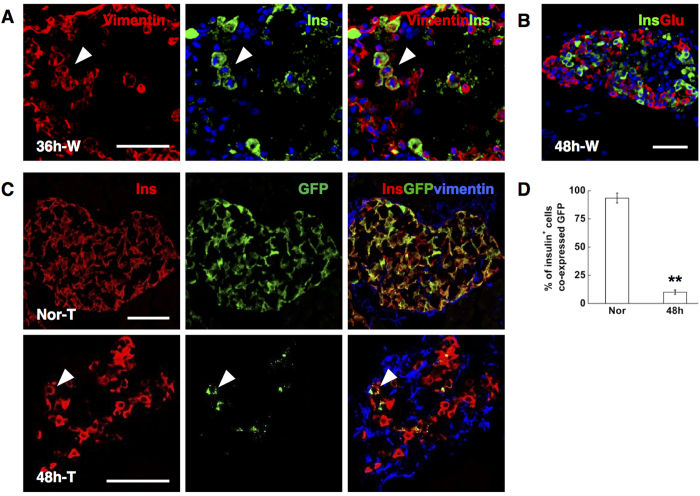
Conditional beta-cell lineage tracing. (**A**) Representative islets stained with antibodies against insulin (green) and vimentin (red) of wild-type mice at 36 hrs post STZ injection (36h-W). Nuclei were labeled with DAPI. Scale bars, 50 μm. The isosceles triangle: round-shaped vimentin^+^ cells gaining insulin expression at 36 hrs. (**B**) Photomicrographs double stained with anti-insulin (green) and anti-glucagon (red) antibodies of wild-type mice at 48 hrs after STZ injection (48h-W). Nuclei were labeled with DAPI. Scale bars, 50 μm. (**C**) Representative islets stained with antibodies against insulin (red) and vimentin (blue) of transgenic mice at 48 hrs post STZ injection (48h-T). The isosceles triangle: insulin^+^ cells showing GFP expression. Scale bars, 50μm. (**D**) Proportion of GFP^+^ beta-cells, as a percentage of insulin^+^ cells. Data are shown as means ± SEM, n = 3 per group; **P < 0.01.

Using genetic cell-lineage tracing, beta cells cultured from adult human islets were shown to undergo rapid dedifferentiation with a rapid loss of beta-cell and epithelial phenotype, and a pronounced increase in levels of transcripts encoding mesenchymal markers like vimentin in parallel^27^. Moreover, the dedifferentiated beta cells can be induced to redifferentiate in culture^28^,^29^. To investigate the possibility of dedifferentiation and redifferentiation of escaping beta-cells in this model, we adopted mice in which the insulin promoter drives the expression of tamoxifen-dependent Cre recombinase^9^ and R26-GFP used as reporter. The administration of tamoxifen induces the expression of the reporter marker GFP from the Rosa26 locus exclusively in beta-cells: roughly all of them (93.5 ± 4.4%) were GFP^+^ (Fig. 5D). In wild-type mice, round-shaped vimentin/insulin double positive cells were observed at 36 hrs after a single high dose of STZ (200 mg/kg) treatment (Fig. 5A), giving rise to beta-cell regeneration at 48 hrs (Fig. 5B). Therefore the proportion of insulin^+^ cells labeling GFP was determined in transgenic mice at 48 hrs to measure the contribution of surviving beta-cells to regeneration. Forty-eight hours after ablation, the number of GFP^+^ cells was extremely rare, indicating a nearly complete destruction of original beta-cells. Furthermore, the proportion of insulin-producing cells bearing GFP expression plummeted to 10.0 ± 2.0% (Fig. 5C,D). The dilution of labeled beta-cells suggested that the regeneration was from heterologous, that is, non beta-cell, origins.

### The vimentin^+^ cells did not express nestin nor marker of macrophages

Beta-cell damage induced by a single high dose of STZ is followed by macrophage recruitment^14^, as shown in Supple-mentary Fig. 1B, which is necessary for limiting the area of tissue damage, and for cleansing cell debris. As macrophages are reported to express vimentin^30^, we set out to determine if the insulin expressing vimentin^+^ cells were macrophages. Control pancreas showed a few resident macrophages outside the islets. At 16 hrs, there was a significant increase in F4/80^+^ cells, which located around the islet exterior, while the round-shaped vimentin^+^ cells predominantly distributed in the core area of the islets. Moreover, the infiltrating macrophages showed irregular shapes. At 24 hrs, when the round-shaped vimentin^+^ cells strongly expressed insulin within the islet, withdraw of the macrophages was observed with the number and distribution of macrophages showed no difference with the normal control (Supplementary Fig. 3). Thus, we excluded macrophages as the origin of the group of round-shaped vimentin^+^ cells.

Nestin is a key surface marker of neuronal and haematopoietic stem cells^31^. Previous studies also identified nestin-expressing cells in the islets as potential pancreatic progenitor cells^32^. We therefore assessed changes in nestin expression in the pancreas. No recruitment of nestin^+^ cells were detected during the regenerative process and the round-shaped vimentin^+^ cells did not co-express nestin (Supplementary Fig. 4), ruling out a contribution of nestin^+^ cells to beta cell regeneration in our model.

### The vimentin^+^ cells did not arise predominantly from Ngn3^+^ progenitor cells

During development, endocrine cell differentiation involves the EMT (epithelial-to-mesenchymal transition) of Ngn3^+^ endocrine progenitor cells^33^, generating cells possessing mesenchymal features. In addition, the reactivation of Ngn3^+^ progenitor cells has been testified as giving rise to beta-cell regeneration in adults after pancreatic duct ligation (PDL)^7^. To investigate the participation of Ngn3^+^ endocrine progenitors, we assessed the mRNA level of Ngn3 during the analysis period. A 30-fold increase of Ngn3 transcripts was observed 24 hrs following STZ-treatment (Supplementary Fig. 5A). As well known, Ngn3 expression in normal adult rodents was hardly detectable. In addition, previous study reported hundreds of times higher expression of Ngn3 after PDL. So we postulated that a 30-fold increase of transcripts might be the result of activation of a very small number of progenitor cells. Indeed, immunostaining of Ngn3 showed that less than three Ngn3 positive cells could be detected per section (Supplementary Fig. 5B). Moreover, very few of the Ngn3^+^ cells were co-expressed with vimentin (Supplementary Fig. 5B). Also, since Ngn3 induces the EMT during pancreas morphogenesis^34^,^35^, the Ngn3 activation should have been prior to the emergence of the group of vimentin^+^ cells, which was not the case in our model. In conclusion, the group of vimentin positive cells was unlikely to arise from Ngn3^+^ progenitors that underwent EMT, although we can’t exclude the possibility that Ngn3^+^ progenitors might contribute to a small population of newly formed beta-like cells.

### The insulin producing vimentin^+^ cells showed strong MafB expression

Considering that the alpha- to beta-cell conversion has been reported following extreme beta-cell loss in adult mice, we hypothesized that the typically round-shaped vimentin^+^ cells might originate from alpha-cells.

**Figure 6:**
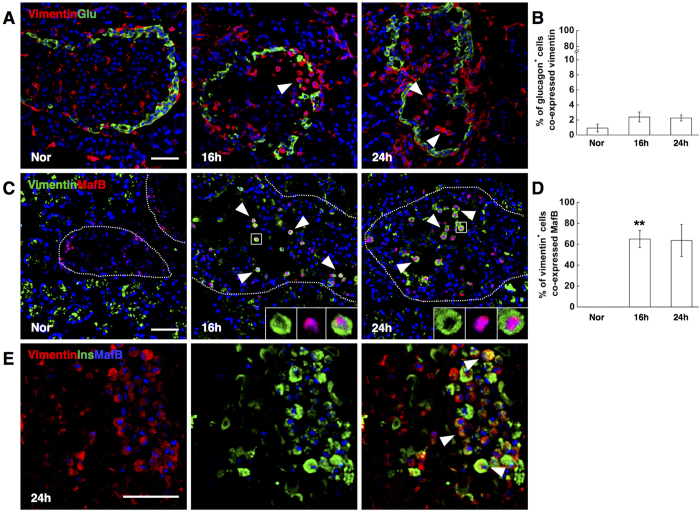
Round-shaped vimentin^+^ cells showed strong MafB expression. (**A**) Photomicrographs double stained with anti-vimentin (red) and anti-glucagon (green) antibodies. Nuclei were labeled with DAPI. Scale bars, 50 μm. The isosceles triangle: round-shaped vimentin^+^ cells showing negative glucagon expression. (**B**) Quantification of glucagon-positive cells co-expressing vimentin. (**C**) Photomicrographs double stained with anti-vimentin (green) and anti-MafB (red) antibodies. Nuclei were labeled with DAPI. Scale bars, 50 μm. The isosceles triangle: round-shaped vimentin^+^ cells coexpressing MafB. The boxed regions showed the high powered photomicrograph at the bottom right of each image. (**D**) Quantification of vimentin-positive cells co-expressing MafB. (**E**) Photomicrographs triple stained with anti-vimentin (red), anti-insulin (green) and anti-MafB (blue) antibodies. Scale bars, 50 μm. The isosceles triangle: cells co-expressing vimentin, insulin and MafB. Data are shown as means ± SEM, n = 5–6 per group. *P < 0.05 and **P < 0.01.

Glucagon/vimentin double staining was first performed to test this possibility. Unexpectedly, rare vimentin^+^ cells were found to co-express glucagon from 16 hrs to 24 hrs after STZ injection (Fig. 6A,B). Inspiringly when we next chose the transcription factor MafB as the alpha-cell indicator, which is restricted to alpha-cells within the adult pancreas^36^,^37^, a surprisingly high percentage of the round-shaped vimentin^+^ cells in the islets co-expressed MafB from 16 hrs to 24 hrs post STZ injection (Fig. 6C,D). In addition, the staining of MafB in vimentin^+^ cells was even more intense than in vimentin negative alpha-cells. Vimentin/insulin/MafB triple staining at 24 hrs following STZ injection further confirmed that almost all the vimentin^+^/insulin^+^ cells coexpressed MafB (Fig. 6E). Taken together, our data indicated that a group of round-shaped vimentin^+^/MafB^+^/glucagon^−^ cells could serve as beta-cell progenitors after STZ-induced extreme beta-cell loss.

## Discussion

Our study demonstrated that extreme beta-cell ablation induced by a single high dose of STZ in adult rats triggered a group of round-shaped vimentin^+^/MafB^+^/glucagon^-^ cells, which adopted insulin production, further differentiated into beta-like cells, and acquired functionality over time if blood glucose level was well controlled. Our research identified a novel intermediate cell type that served as beta-cell precursor after a single-high dose of STZ-induced massive beta-cell injury and provided new insight into beta-cell regeneration.

Based on current data, it is hard to make an assertion about the origin of the vimentin^+^/MafB^+^ intermediate cells. Yet alpha-cells seem to represent a viable postulation. First, according to previous reports, the amount of beta-cell loss influences the degree of cell plasticity and regenerative resources of the adult pancreas^3^. Beta-cell loss must be near total for triggering alpha- to beta-cell conversion^8^,^38^. Our dosage of STZ resulted in an elimination of more than 99% of beta-cells, which logically could be a proper ablation extent to enhance alpha-cell plasticity and induce alpha- to beta- conversion. Second, MafB is exclusively expressed in alpha-cells in adult pancreas^37^. Previously, high incidences of glucagon/insulin co-expressing cells (30–60% of the emerging insulin^+^ cells) were reported to accompany the alpha- to beta- transition, while in our study the intermediate cells did not show glucagon expression^8^,^38^. But we noticed that during delta- to beta- conversion in juvenile mice, the process involved dedifferentiation of the starting cells with loss of somatostatin expression, leading to the absence of insulin somtostatin co-expression during the transdifferentiation^12^. Therefore, the absence of glucagon/insulin co-expression could not negate the possibility of alpha- to beta-cell conversion in our model. Of course, based on current data, we were indeed not able to exclude the possibility that observed beta-cells were originated from a group of unknown mesenchymal cells, which gained MafB and subsequently, insulin expression. After all, marker colocalization per se is not proof of ontogenetic relationships between different cell types and a lineage tracing study is indispensable to identify the origin of the vimentin^+^/MafB^+^ cells.

Beta-cell-enriched transcription factors, such as Pdx1 and Nkx6.1, play crucial roles in maintaining and inducing beta-cell identity. Pdx1 binds directly to insulin promoters, thus inducing insulin transcription^39^. Ectopic Pdx1 expression, alone or combined with other factors, drives hepatocytes or acinar cells into insulin-producing cells^10^,^40^. Nkx6.1 also contribute to the activation of beta-cell-specific genes^41^. However, these beta-cell-specific genes were not activated in newly-formed insulin-producing cells at 24 hrs after STZ treatment, indicating that the insulin production at this time point appears to be independent of beta-cell-specific transcription factor activity. MafB, which contributes to expression of the glucagon gene in adult alpha-cells, is also involved in regulating key genes required for beta-cell differentiation during development and is capable of activating insulin expression *in vitro*^37^,^42^. Hence we postulate that the highly activated MafB might directly induce the transcription of the insulin gene 24 hrs following STZ treatment.

After injury, resident tissue macrophages are supplemented by an active recruitment of blood monocytes, which then differentiate into macrophages and dendritic cells. Macrophages are abundant during all stages of tissue repair and have an important influence on the progress and resolution of tissue damage. Accumulating data suggest that macrophages produce different mediators during normal regeneration after injury in several organs, such as liver, kidney, and heart^43^,^44^,^45^. In addition, recent reports indicate that macrophages produce cell type–specific signals required for pancreatic cell regeneration in mice after extensive ablation of both acinar and endocrine cells^46^. In our model, the injection of STZ leads to rapid cell death of the beta-cells, followed by massive infiltration of macrophages. Although macrophages did not contribute directly to beta-cell regeneration in the model, whether macrophages contribute to be beta-cell regeneration by providing the proper signals to generate the group of vimentin^+^ cells needs to be addressed.

## Materials and Methods

### Animals and Treatment

Eight-week-old male Sprague-Dawley (SD) rats (Laboratory Animal Research Center of the Academy of Military Medical Science) weighing 240–260 g were injected intraperitoneally with a single dose of STZ (65 mg/kg, Sigma, St Louis, USA). STZ was dissolved in 0.05 M citrate buffer (pH 4.5) and injected within 15 minutes of preparation. Forty-eight hours post STZ injection the rats were randomly treated with: no treatment (referred as non-treated rats), and daily subcutaneous injection of glargine insulin, a long-acting human insulin analogue, that restore normoglycemia (BG <11.1 mM, referred as insulin-treated rats) for 7 days. Normal rats of the same age were used as normal control. IPGTTs, IRTs and GSIS were performed in normal control, at 48 hrs after STZ treatment, and after 7 days’ insulin treatment with one-day washout in ins-treated and concurrent non-treated control rats. GTT and IRT were performed by injecting 2 g/kg of D-Glucose IP after fasting for 12 hrs. Glucose levels and serum insulin levels were detected at 0, 30, 60, 90, 120 minutes after glucose injection. For *in vitro* GSIS, isolated islets (120 ~ 150 islets from 6 rats of each group) were pretreated in 12-well plates in low-glucose buffer (1.7 mM) for 1 h, followed by sequential treatment with low-glucose solution (1.7 mM) for 1 h and high-glucose solution (20.2 mM) for 1 h. The buffer for incubation was Krebs Ringer Bicarbonate HEPES Solution (KRB). Then the media at low and high glucose levels were collected and analyzed by ELISA (Rat Insulin Elisa Kit; Millipore, St. Charles, MO). The results were presented by the fold change of insulin release to assess glucose responsiveness.

We adopted mice in which the insulin promoter drives the expression of tamoxifen-dependent Cre recombinase (RIP–CreER) and R26-GFP was used as reporter. Tamoxifen was freshly prepared (50 mg/ ml; Sigma) and administered with a gastric catheter (five doses of 10 mg, every 2 days) to induce GFP activity. Then eight-week-old transgenic male mice were injected intraperitoneally with a single dose of STZ (200 mg/kg, Sigma) for further study. All animal procedures were approved by the Institutional Animal Care and Use Committee of the Chinese PLA general hospital and carried out in accordance with the guidelines of China Council on Animal Care and Use.

### Preparation of Tissue Samples

The rats, wild type mice or the transgenic mice, which were sacrificed at indicated time points, were injected intraperitoneally with 1% Pentobarbital Sodium (50 mg/kg) and then perfused through the left ventricle with 100/10 ml PBS, followed with 500/50 ml 4% paraformaldehyde. When the perfusion finished, the pancreas were isolated and incubated in 30% sucrose/PB overnight. The tissues were then embedded (Tissue-Tek OCT Compound; Sakura Finetek, Torrance, CA) and frozen at −80 °C for long-term storage.

### Histology and Immunohistochemistry

For the histology of the pancreas, paraffin sections of 5 μm were stained with hematoxylin and eosin (Richard Allan Scientific, Kalamazoo, MI). For the immunofluorescence analysis, the frozen sections were incubated for 14 hours at 4 °C with antisera specific for insulin (1/150, guinea pig, Sigma), glucagon (1/2,000, mouse, Sigma), E-cadherin (1/100, rabbit, Abcam, San Francisco, USA), vimentin (1/100, rabbit, Abcam), vimentin (1/100, mouse, Sigma), MafB (1/200, rabbit, Bethyl Laboratories, Montgomery, USA), MafA (1/200, rabbit, Bethyl Laboratories), Pdx1 (1/50, goat, R&D system, Minneapolis, USA), Ngn3 (1/200, rabbit, Millipore), F480 (1/200, rabbit, Santa Cruz, Texas, USA), nestin (1/200, mouse, Abcam) and Ki67 (1/50, mouse, BD, San Diego, CA ). The slides were then incubated for 2 hours at room temperature with species-specific secondary antibodies (1:500; Alexa-594, Alexa-488 or Alexa-405; Invitrogen, Basel, Switzerland). The nuclei were visualized with DAPI (40, 6-diamidino-2-phenylindole) (Sigma). Images were captured with a Fluoview FV1000 camera (Olympus, Tokyo, Japan) and recorded on a computer using the Olympus Fluoview Ver.1.7a viewer.

### Quantitative RT-PCR

Total RNA extraction, cDNA synthesis and Q-PCR: Adult pancreata (n = 3 rats/time-point) were harvested at 5 time-points during regeneration (normal, 8 hrs, 16 hrs, 24 hrs). Adult islets were isolated as described^47^ and pooled from 5 rats for each time-point (normal, 16 hrs, 24 hrs and 48 hrs). RNA samples were extracted from tissues or isolated cells using the TRIzol reagent (Invitrogen, Carlsbad, CA). Single-stranded cDNA was synthesized using SuperScript II reverse-transcriptase and oligo (dT) (Invitrogen, USA). Real-time PCR analysis was performed using Power SYBR Green RT-PCR Reagent (Applied Biosystems, Carlsbad, CA) on ABI Prism thermal cycler model StepOnePlus (Applied Biosystems, Carlsbad, CA). The thermal cycling program was 50°C for 2 minutes, followed by 95°C for 10 minutes for 1 cycle, then 95°C for 30 seconds, followed by 60°C for 1 minute for 40 cycles. Melting curve analysis was performed to ensure the specificity of primers. Beta-actin was used as a reference gene in each reaction. The PCR primers were listed in Table 1.

### Morphologic analysis and quantifications

The pancreas samples were sectioned at 7 μm. Fifty consecutive sections were performed from each pancreas sample. Following the immunofluorescence staining of serial sections, the number of different cell types in each islet was manually counted in the islet microscopy images. For each cell type, 40–50 representative islets from 5–6 rats were counted. For transgenic mice, 15–20 representative islets from 3 mice were counted.

### Statistical Analyses

All data are presented as means ± S.E.M. The statistical analysis was performed using the SPSS v.14.0.1 software for Windows. Differences between means were assessed using a Student’s *t* test, Chi-square test or by one-way ANOVA when required. Group differences at the level of P < 0.05 were considered statistically significant.

## Additional Information

**How to cite this article**: Cheng, Y. *et al.* Beta-cell regeneration from vimentin^+^/MafB^+^ cells after STZ-induced extreme beta-cell ablation. *Sci. Rep.* **5**, 11703; doi: 10.1038/srep11703 (2015).

## Supplementary Material

Supplementary Information

## Figures and Tables

**Table 1:** List of sequences of forward and reverse primers.

Genes	Primer Sequences	Product size (bp)
Ins1	Forward: 5′->3′ggaacgtggtttcttctacac Reverse: 5′->3′gggagtggtggactcag	216
Ins2	Forward: 5′->3′tcttctacacacccatgtccc Reverse: 5′->3′ggtgcagcactgatccac	149
Pdx1	Forward: 5′->3′gacacatcaaaatctggttccaaa Reverse: 5′->3′tcccgctactacgtttcttatcttc	75
MafA	Forward: 5′->3′cttcagcaaggaggaggtcatc Reverse: 5′->3′gcgtagccgcggttctt	67
Nkx6.1	Forward: 5′->3′tcttctggcctggggtgatg Reverse: 5′->3′ggctgcgtgcttctttctcca	121
Glut2	Forward: 5′->3′tgggttccttccagttcg Reverse: 5′->3′aggcgtctggtgtcgtatg	166
Ngn3	Forward: 5’->3′tggcactcagcaaacagcga Reverse: 5’->3’acccagagccagacaggtct	101
Beta-actin	Forward: 5′->3′tggcactcagcaaacagcga Reverse: 5′->3′acccagagccagacaggtct	101
