# Supplementary material for: Beta-cell regeneration from vimentin+/MafB+ cells after STZ-induced extreme beta-cell ablation
Source: Sci Rep. 2015 Jul 1;5:11703. doi: 10.1038/srep11703 (PMC4486952; doi:10.1038/srep11703)
Supplement: Supplementary Information [file srep11703-s1.pdf]

Beta-cell regeneration from vimentin$^+$/MafB$^+$ cells after STZ-induced extreme beta-cell ablation

Yu Cheng[a,b,e], Hongjun Kang[d,e] Jing Shen[a,b,c,e], Haojie Hao[b], Jiejie Liu[b], Yelei Guo[b], Yiming Mu[a]* and Weidong Han[b]*

[a]*Department of Endocrinology, Chinese PLA General Hospital, 28 Fuxing Road, Beijing 100853, China;*[b]*Department of Molecular Biology, Institute of Basic Medicine, School of Life Science, Chinese PLA General Hospital, 28 Fuxing Road, Beijing 100853, China;* [c]*Department of Endocrinology, Chinese PLA 309 Hospital, 17 Heishanhu Road, Beijing 100091, China;* [d]*Department of Critical Care Medicine, Chinese PLA General Hospital, 28 Fuxing Road, Beijing 100853, China.*

[e]*These authors contributed equally to this work*

*Correspondence should be addressed to:
MD., Ph.D. Yiming Mu
Department of Endocrinology,
Chinese PLA General Hospital,
Beijing 100853, China
E-mail: muyiming@301hospital.com.cn
Tel.: +86(10)55499001
Fax: +86(10)68168631

MD., Ph.D. Weidong Han
Department of Molecular Biology,
Institute of Basic Medicine,
School of Life Sciences,
Chinese PLA General Hospital,
Beijing 100853, China
E-mail: hanwdrsw69@yahoo.com
Tel.: +86(10)66937463
Fax: +86(10)66937516

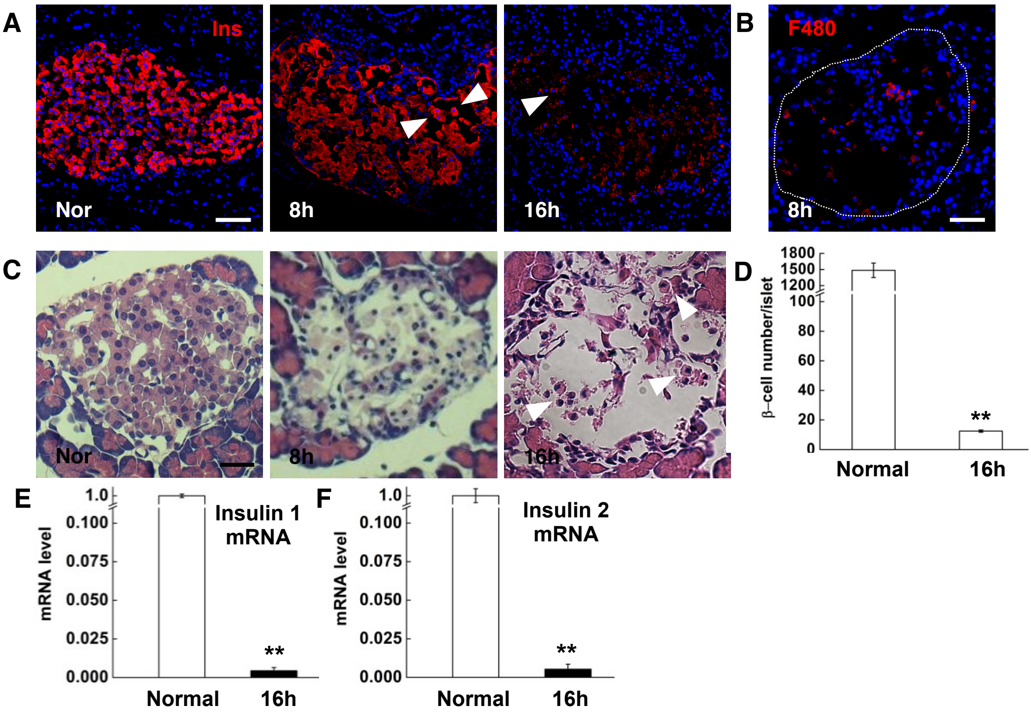

Figure S1. A single high dose STZ induced acute and extreme beta-cell injury. (A) Representative islets stained with insulin (red) of normal control SD rats (Nor), and STZ-treated rats at indicated time points (8h, 16h). Nuclei were labeled with DAPI. Scale bars, 50µm. The isosceles triangle: remaining insulin stained cells. (B) Representative islets stained with F480 (red) of STZ-treated rats (8h). Scale bars, 50µm. (C) Representative islets from rat pancreas sections (4µm). The sections were performed with hematoxylin and eosin staining. The isosceles triangle: a group of round-shaped cells. Scale bars, 20µm. (D) Quantification of b-cell number. (E, F) Ins1 and Ins2 gene expression in isolated islets of normal control and STZ-treated rats (16h). Data are shown as means ± SEM, n=5-6 rats per group. $^{*}P < 0.05$ and $^{**}P < 0.01$.

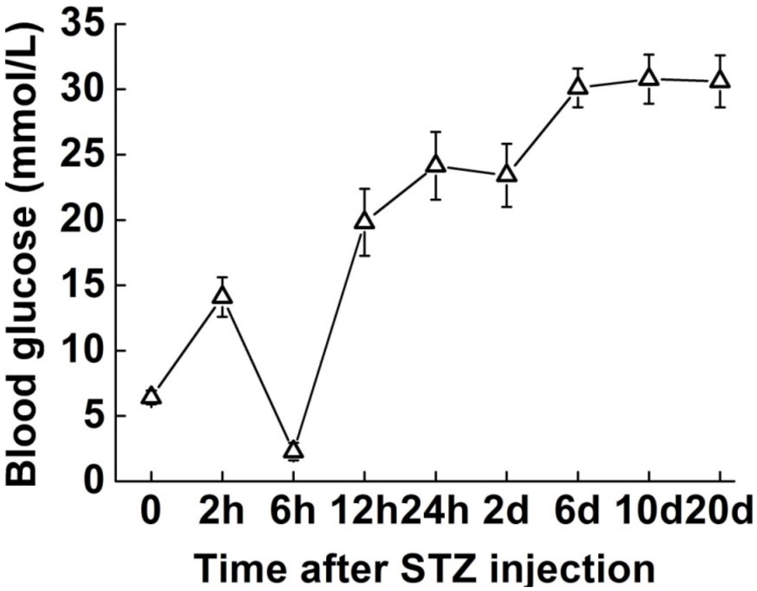

Figure S2. Random blood glucose levels post STZ injection. Data are shown as means ± SEM, n=10 rats.

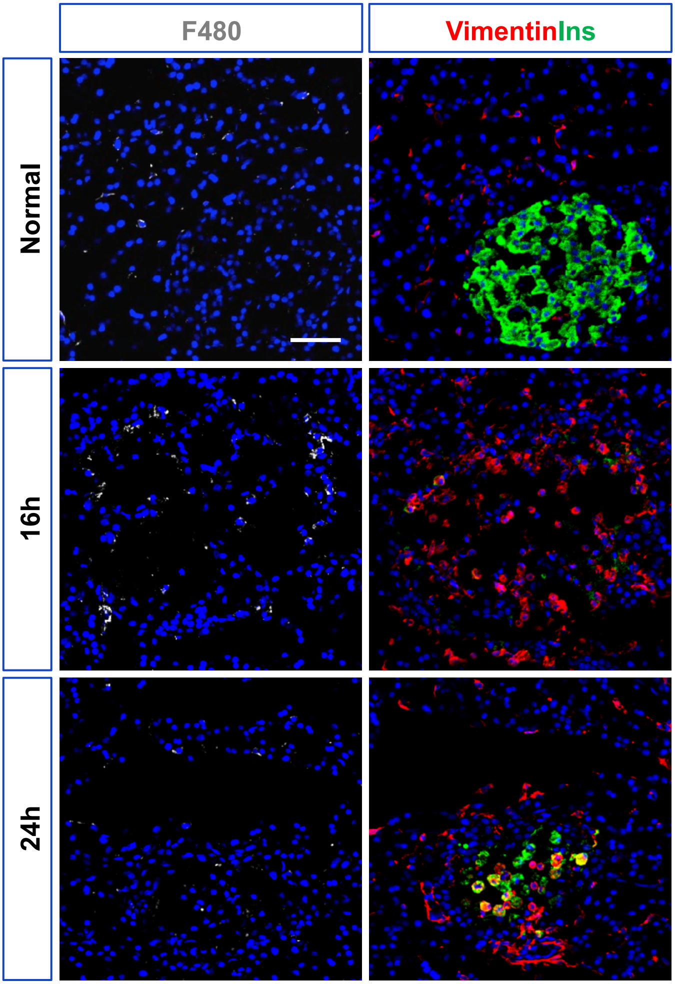

Figure S3. The round-shaped vimentin⁺ cells did not express macrophage marker F480. The photomicrographs in the left column showed representative islets stained with anti-F480 (gray), and the right column with anti-vimentin (red) and anti-insulin (green) antibodies of the consecutive slides from normal control SD rats (Nor), and STZ-treated rats at different time points (16 h, 24 h). Nuclei were labeled with DAPI. Scale bars, 50μm.

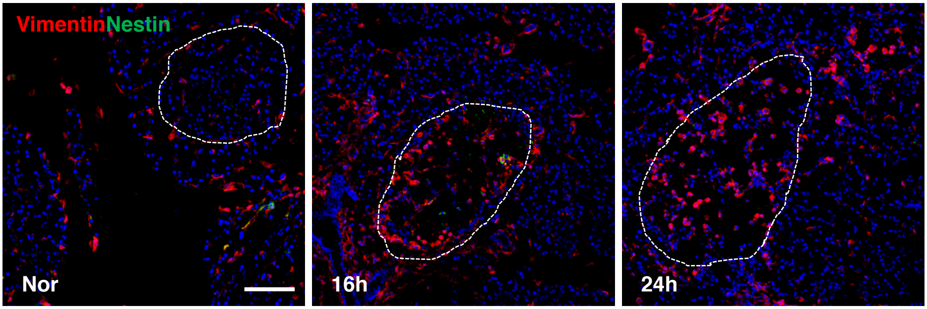

Figure S4. Very limited recruitment of nestin-positive cells to damaged islets. The photomicrographs stained with anti-vimentin (red) and anti-nestin (green) antibodies from normal control SD rats (Nor), and STZ-treated rats at different time points (16 h, 24 h). Nuclei were labeled with DAPI. Scale bars, 100μm.

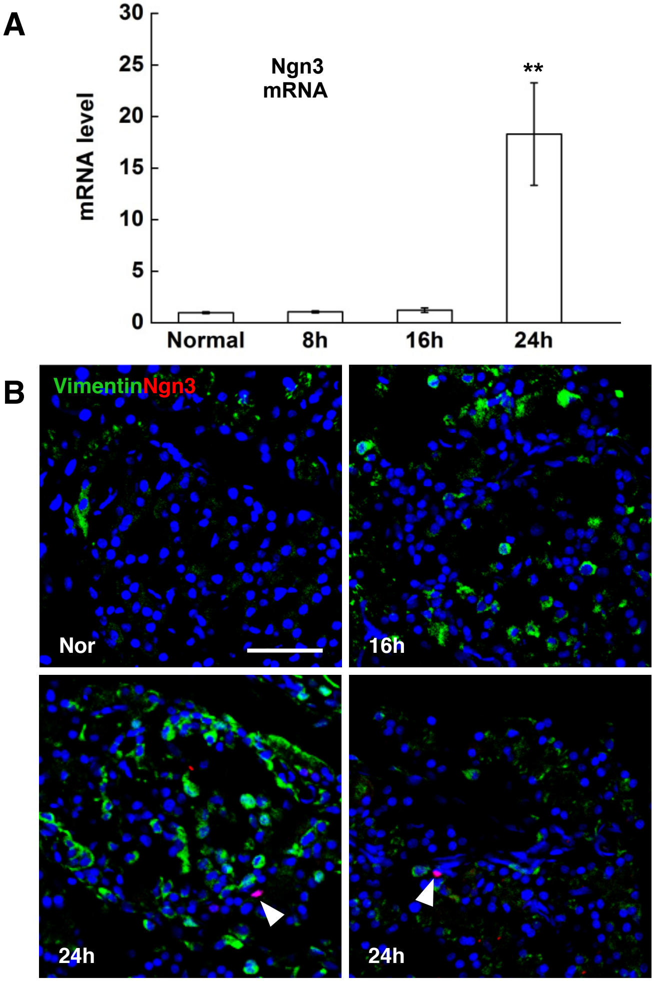

Figure S5. Massive beta-cell loss induced limited Ngn3 activation. (A) Expression of Ngn3 gene in pancreas of normal control and STZ-treated rats at indicated time points (8 h, 16 h, and 24 h). (B) Photomicrographs double stained with anti-vimentin (green) and anti-Ngn3 (red) antibodies of normal control and STZ-treated rats (16 h, 24 h). Nuclei were labeled with DAPI. Scale bars, 50μm. The isosceles triangle: $Ngn3^{+}$ cells. Data are shown as means ± SEM, n=3 per group; *$P < 0.05$ and **$P < 0.01$.
